# Transcriptomics and Fitness Data Reveal Adaptive Plasticity of Thermal Tolerance in Oysters Inhabiting Different Tidal Zones

**DOI:** 10.3389/fphys.2018.00825

**Published:** 2018-08-20

**Authors:** Ao Li, Li Li, Wei Wang, Kai Song, Guofan Zhang

**Affiliations:** ^1^Key Laboratory of Experimental Marine Biology, Institute of Oceanology, Chinese Academy of Sciences, Qingdao, China; ^2^University of Chinese Academy of Sciences, Beijing, China; ^3^Laboratory for Marine Fisheries and Aquaculture, Qingdao National Laboratory for Marine Science and Technology, Qingdao, China; ^4^National and Local Joint Engineering Key Laboratory of Ecological Mariculture, Institute of Oceanology, Chinese Academy of Sciences, Qingdao, China; ^5^Laboratory for Marine Biology and Biotechnology, Qingdao National Laboratory for Marine Science and Technology, Qingdao, China

**Keywords:** adaptive divergence, evolutionary trade-offs, global warming, oyster, phenotypic plasticity, fitness-related trait, transcriptomic

## Abstract

Fine-scale adaptive evolution is always constrained by strong gene flow at vertical level in marine organisms. Rapid environmental fluctuations and phenotypic plasticity through optimization of fitness-related traits in organisms play important roles in shaping intraspecific divergence. The coastal systems experience strong variations in multiple abiotic environmental factors, especially the temperature. We used a typical intertidal species, Pacific oyster (*Crassostrea gigas*), to investigate the interaction between plasticity and adaptive evolution. We collected intertidal and subtidal oysters from two ecological niches and carried out common garden experiments for one generation. We identified fine-scale vertical adaptive divergence between intertidal and subtidal F_1_ progeny at both sites, based on different hierarchical phenotypes, including morphological, physiological, and molecular traits. We further quantified the global plasticity to thermal stress through transcriptomic analysis. The intertidal oysters exhibited slow growth rate. However, they showed high survival and metabolic rates under heat stress, indicating vertically fine-scale phenotypic adaptive mechanisms and evolutionary trade-offs between growth and thermal tolerance. Transcriptomic analysis confirmed that the intertidal oysters have evolved high plasticity. The genes were classified into three types: evolutionarily divergent, concordantly plastic, and adaptive plastic genes. The evolved divergence between intertidal and subtidal oysters for these gene sets showed a significant positive correlation with plastic changes of subtidal populations in response to high temperature. Furthermore, the intertidal oysters exhibited delayed large-scale increase in expressional plasticity than that in subtidal counterparts. The same direction between plasticity and selection suggests that the oysters have evolved adaptive plasticity. This implies that adaptive plasticity facilitates the oyster to adapt to severe intertidal zones. The oysters exposed to strong environmental variability are thermal tolerant and have high adaptive potential to face the current global warming. Our findings will not only provide new insights into the significant role of plasticity in adaptive evolution that can be extended to other marine invertebrates, but also provide basic information for oyster resources conservation and reef reestablishment.

## Introduction

Understanding the responses of organisms to stressful or novel environments with strong selection pressures is one of the long-standing problems in evolutionary biology. Furthermore, adaptive evolution driven by high degree of environmental variability, currently a major threat to global biodiversity (Bay et al., 2017), has gained attention recently (Sanford and Kelly, 2011; Vargas et al., 2017). The coastal zones experience strong spatial (tidal level) and temporal (seasonal and daily) environmental fluctuations, such as temperature and exposure time to extreme low tide. Organisms experience high variations in temperature fluctuation at different intertidal zones that are just a few meters apart (Gracey et al., 2008), which provide interesting models to investigate environment-induced adaptive evolution in response to exacerbated global climate change (Zhang et al., 2012; Vargas et al., 2017). Fine-scale vertical adaptive divergence has been reported in many marine organisms inhabiting high and low intertidal zones (Petes et al., 2008; Place et al., 2012; Bedulina et al., 2013) despite high gene flow in some species with long-time planktonic larval period (Bozinovic et al., 2011; Sanford and Kelly, 2011). Furthermore, studies have reported that intertidal organisms have evolved higher phenotypic plasticity than the subtidal counterparts in order to adapt to fluctuating environments (Somero, 2012; Kenkel et al., 2013; Kenkel and Matz, 2016). Phenotypic plasticity refers to the ability of a single genotype to produce different phenotypes in response to variable environmental gradients (Pfennig et al., 2010). However, systematical investigations of vertically fine-scale adaptive evolution are rarely reported by integrating fitness-related traits and omic analysis.

The phenotypes influencing the fitness of an individual are recognized as adaptive traits. Variations in fitness-related morphological and physiological traits along spatial and temporal scales are considered important aspects to assess the evolutionary potential of intraspecific populations inhabiting variable environmental gradients (Bozinovic et al., 2011; Somero, 2012). However, the importance of phenotypic mechanisms to adaptive evolution has been underestimated due to random noise. Physiological responses of organisms to environmental changes are particularly important given the current scenario of global warming. In addition to the fitness-related trait of growth (Hice et al., 2012; Burford et al., 2014), the responses to acute thermal stress under sublethal and extreme temperatures have been frequently used to evaluate temperature adaptation at whole-organism, cellular, and biochemical levels (Somero, 2012; Yampolsky et al., 2014). Furthermore, in recent studies, molecular phenotypes (such as gene expression) are being increasingly used to identify adaptive divergence in response to a wide range of abiotic stressors, especially temperature (Somero, 2012; Bedulina et al., 2013; Kenkel et al., 2013; Dayan et al., 2015). Whole-genome gene expression profile is the proximate mechanism that links genotype to phenotype. It has been hypothesized to be of adaptive importance in providing raw material for the evolution of adaptive phenotypic plasticity. Comparative transcriptional analysis can accurately quantify the ability of plasticity among populations dwelling in different environments (Fischer et al., 2016; Kenkel and Matz, 2016). The organisms inhabiting highly fluctuating environments alternatively enhance their evolutionary responses by typically evolving adaptive phenotypic plasticity (Scoville and Pfrender, 2010; Nadeau et al., 2017). Furthermore, molecular phenotypes can be efficiently used to identify the direction of plasticity and evolution (adaptive or non-adaptive, Fischer et al., 2016). Both play important roles in adaptive evolution; however, adaptive plasticity potentiates evolutionary rescue by increasing the survival of organisms, especially those dwelling in heterogeneous environments (Markov and Ivnitsky, 2016). Thus, it can buffer the effects of climate change and provide more time for evolution. Furthermore, it will help evaluate the evolutionary potential of a species confronting aggravated environmental challenges, especially non-traditional model organisms, such as marine invertebrates.

Oysters have successfully colonized the estuaries and coastal zones, and offer diverse ecological services. Oyster reefs are vital for the health of the coast because they effectively protect shorelines, provide habitat and food for numerous species, and improve the quality of water. Degradation of this suspension-feeding oyster reef community might predispose estuaries to increased environmental deterioration. About 85% of oyster reefs have already been lost globally, and even worse, more than 90% of oyster reefs are lost along the northern coasts of China due to anthropogenic activities and climate change (Beck et al., 2011). Furthermore, the global average warming in the surface temperature of sea over the past half century is 0.11°C per decade, and one of the warming maximum is present around 40° of the Northern Hemisphere (Rhein et al., 2013). Effective protection and reestablishment of oyster reefs are of great importance, which require deep characterization of the existed genetic resources.

Asia Pacific is believed to be the epicenter of oyster speciation because of the presence of a large number of oyster species (Ren et al., 2010). The Pacific oyster, native to Northeastern Asia, is a naturally dominant species of the northern coast of China (Ruesink et al., 2005). It is used as a model species to study intertidal adaptation (Somero, 2012; Zhang et al., 2012; Guo et al., 2015). In the present study, we investigated fine-scale vertical adaptive divergence with the role of plasticity in adaptive landscape as the supplement (from our previous study) focusing on regional divergence (Li et al., 2018) in oyster populations from different latitudes. The intertidal and subtidal oysters from two sampling sites of the Bohai Sea, within the warming sensitive zone, were collected and common gardened for one generation to potentially minimize the effects of environment (Sanford and Kelly, 2011). We aimed to examine adaptive divergence at vertical level and the role of plasticity in adaptive evolution by specific phenomic and transcriptomic analyses. Understanding the responses of adaptive traits and plastic changes to environmental factors, especially high temperature, will (1) help understand the interaction between phenotypic plasticity and adaptive evolution in the marine organism, (2) help forecast adaptive potential of oyster populations (including other coastal animals) inhabiting different ecological niches facing rapid global climate change, and (3) provide insights into the oyster resource conservation and reef reestablishment, which will ultimately advance effective management of marine ecosystem.

## Materials and Methods

### Sampling and Common Garden Experiments

Four wild oyster populations were collected from the intertidal (I) and subtidal (S) zones of two latitudinal sites [Bayuquan (BYQ): 40°18′N and Laoting (LT): 39°16′N] and translocated to Qingdao for 1-month acclimation before breeding (**Supplementary Figure 1**). Common garden experiments were conducted according to the methods of Li et al. (2017) (**Supplementary Figure 2**). Eggs from 30 uncontaminated mature female oysters of each population were mixed and divided into 30 replicates. Each replicate was fertilized individually with the sperms from 30 mature male oysters. The zygotes were incubated in a 70L plastic container. At the D-shaped stage, six containers were combined to form one group with three culture replicates. All culture replicates were well maintained in the sea. Before the experiments, the F_1_ generation oysters were acclimatized in the laboratory aquarium (temperature: 14.2–18.2°C; salinity: 31.3 ± 0.5‰) for 2 weeks with aerated, filtered seawater. The oysters were fed commercial spirulina powder. The seawater was changed every day during this period.

Furthermore, satellite remote sensing data of monthly average sea surface temperature (SST) within 30 km from each sampling site during the last 18 years were collected from 2000 to 2017^1^. The time of extreme low tide and the corresponding tidal level during day time in the warmest month (August) were recorded because of their prominent effects on the thermal condition of intertidal zones (Helmuth et al., 2002).

### Specific Phenomics

#### Growth

A total of 123–150 oysters were collected from each population. The shell height and wet weight of 4- and 10-month-old oysters were measured after cleaning the epibionts.

#### Metabolites

The soft tissue of fifty 11-month-old oysters from each population were sampled, immediately frozen in the liquid nitrogen, and stored at -80°C. The tissues were then freeze-dried for 48 h and individually ground to powder. For each population, five samples were obtained by mixing equal amounts of powder of 10 oysters. The metabolites including total protein, glycogen, and fat were determined by near infrared reflectance spectroscopy.

#### Survival

We examined the thermotolerance of 4-month-old oysters (*n* = 209–326). The oysters from each population were divided into three groups to determine the survival rate post-acute heat stress of 42°C (LT_50_) for 1 h. During the recovery, the oysters were fed spirulina powder, and the seawater was changed everyday. The shell activity (open or close) of oysters was visually monitored. The oysters whose shell remained open even after touching were considered dead and immediately removed. The survival rate was recorded each of the 14 days following the heat shock.

#### Metabolic Rate

Six 2-year-old oysters from each population were individually placed in a 1.2 L plastic chamber filled with air-saturated seawater at a temperature of 35°C (sublethal temperature) as heat stress. The seawater was stirred slowly using a rotating magnetic stirrer bar beneath the chamber. Needle-type fiber-optic microsensor oxygen optrodes and temperature probe were linked to the oxygen transmitter that simultaneously recorded the temperature and oxygen concentration of the water every 3 s for 1 h. Oxygen consumption of a blank run without the oysters was used to correct the experimental backgrounds. The slope of decrease of oxygen concentration was calculated as the respiration rate (mg. mL^-1^. h^-1^).

#### Heat Stress

Ten-month-old oysters were used to examine the physiological and transcriptional variations in response to heat stress. Previous studies have reported that the sublethal temperature of oysters is 35°C under conditions similar to those used in the present study. The key metabolic transition point from aerobic to anaerobic metabolism ranged from 6 to 24 h (Li et al., 2017). In the present study, the oysters from each population were divided into three replicates and stimulated by high temperature seawater of 35°C for 24 h. The gills of five oysters were individually sampled from each replicate per population at 0, 6, and 24 h (*n* = 15 per sampling time per population). The gills were then flash frozen in liquid nitrogen and stored at -80°C until further processing.

#### Physiological Assessment

Three fundamental physiological indexes, including the content of malondialdehyde (MDA), and the activity of ATPase and superoxide dismutase (SOD), were chosen to indicate the dynamic physiological responses of the oysters to high temperature. Equal amount of frozen gill from five oysters per population was mixed to form biological replicates, and the measurements were taken in duplicates (three biological replicates × two technical replicates per population). The three indexes were measured according to the protocol of the manufacturer (Jiancheng, Nanjing, China). The absorbance value was determined using the Varioskan Flash.

#### Gene Expression

Total RNA was isolated using the RNAprep Pure Tissue Kit (Tiangen, Beijing, China) following the protocol of the manufacturer. The genomic DNA was enzymolysized using DNase I during this procedure. Agarose gel electrophoresis (1.2% gel) and NanoDrop 2000 spectrophotometry were used to test the integrity and concentration of RNA. Equal amounts of 1 μg total RNA from five oysters were mixed per sampling time point within each population (three biological replicates). The cDNA was synthesized using the PrimeScript RT Reagent Kit with gDNA Eraser, and then diluted by 30-fold for quantitative real-time polymerase chain reaction following the procedure of Li et al. (2017). All amplification reactions were conducted in duplicates. Twenty-one heat responsive genes were selected according to previous studies (Zhang et al., 2012; Guo et al., 2015), and the elongation factor 1 alpha (EF 1α) severed as the reference gene. The sequence and amplification efficiency of these genes are presented in the previous study (Li et al., 2017). The relative induced mRNA level was calculated by Livak 2^-ΔΔCT^method, with the gene expression at 0 h as calibrator (Livak and Schmittgen, 2001).

#### Statistical Analysis

All data are presented as mean ± standard error of mean (SEM) and were examined for normality by Shapiro–Wilk test and for homogeneity by Bartlett test. Significant difference in the shell height and wet weight among the four populations was determined by analysis of variance (ANOVA) using the function aov. If the effect of origination of populations was significant, a multiple comparison using the function pairwise.t following Bonferroni *post hoc* test for false discovery rate was performed. The same method was used to test the variation in metabolites and metabolic rate among these populations. The principal component analysis (PCA) was performed to explore the pattern of physiological parameters using the function princomp, and a hierarchical cluster analysis was performed using the pheatmap package for relative gene expression under heat stress. The above statistical analyses were performed using the R software (R Core Team, 2016). The Kaplan-Meier analysis with log-rank test using the SPSS version 19.0 software (IBM SPSS Statistics) was carried out to determine the difference in mortality rate and to estimate survival time in the Survival module. In the present study, the significant level of α = 0.05 was applied.

### Transcriptomic Analysis

#### RNA Sequencing

Transcriptomic analysis was carried out to further investigate the role of plasticity in adaptive evolution globally. The RNA samples used were the same as that of gene expression assessment. Thirty-six mixed RNA samples were used for RNA sequencing. The integrity of RNA was evaluated using the Agilent 2100 Bioanalyzer. The samples with the RNA integrity number (RIN) ≥ 7 were subjected to further analysis. One sample of LT-I_0h_2 was used as an outlier and was abandoned from subsequent analysis. The libraries were constructed using TruSeq Stranded mRNA LT Sample Prep Kit according to the instruction of the manufacturer. These libraries were then sequenced on the Illumina sequencing platform and 125/150 bp paired-end reads were generated. The raw data were processed using the NGS QC Toolkit. The reads containing ploy-N and the low-quality reads were removed to obtain clean reads. The clean reads were then mapped to reference C. gigas genome using Bowtie2. The fragments per kilobase million (FPKM) value of each gene was calculated using cufflinks, and the read counts of each gene were determined by htseq-count.

#### Differentially Expressed Genes

Low-expression genes (those with less than 10 counts in more than 90% of samples)were removed from the dataset. The gene counts were normalized and log-transformed using a regularized log transform with the command rlog() in DESeq2, which provides statistical routines to determine differential expression in digital gene expression data using a model based on negative binomial distribution. The resulting *p*-values were adjusted by the Benjamin and Hochberg approach to control the false discovery rate. The genes with a *p*-value < 0.05 were assigned as differentially expressed.

#### PCA Analysis

We conducted the PCA using the function princomp to visualize overall differences between intertidal and subtidal populations at the two sites using the standardized expression level of each gene.

#### DAPC Analysis

A discriminant analysis of principal components (DAPC) was used to compare the expression of all highly expressed genes using the adegenet package. To compare gene expression plasticity in response to heat stress, we used the unstressed oyster (0 h) axis as a measuring scale to quantify the shift in gene expression in oysters upon heat stress. The magnitude of this shift represents a quantitative measure of genome-wide gene expression plasticity. We inferred the size of these shifts at each sampling time (6 and 24 h) by comparing with that at 0 h (the length of the arrows in **Figure 4** and **Supplementary Figure 4**) using the Markov Chain Monte Carlo (MCMC) linear mixed models. We derived the *p*-value for the population-specific difference by analyzing 2,800 MCMC samples of parameter estimates.

#### Selection of Three Types of Genes

We focused on the selective pressure of high temperature for its critical effects on the intertidal organisms (Somero, 2012). The intertidal populations were considered as warm temperature-adapted counterparts of subtidal oysters. To verify if the genes have significantly evolved in each intertidal population, we restricted the analysis to the samples collected from the oysters that were not subjected to heat stress. The genes that were significantly differentially expressed in oysters from the two sites were considered as evolutionarily divergent genes. Furthermore, the genes exhibiting significant plastic changes in oysters at two stressed times from both intertidal and subtidal populations of the same site were combined because they are potentially derived from the same ancestral population (Sanford and Kelly, 2011). The genes showing significant plastic change at both sites were considered as concordantly plastic genes. In addition, the genes showing significant evolutionary divergence and plastic change were considered as adaptive plastic genes.

#### Relationship Between Plasticity and Divergence

We quantified the plasticity of these three gene sets of the two subtidal oyster populations as difference in the expression values at each sampling time point (6 and 24 h) in contrast to that under ambient condition. We assessed the association between evolved changes (expressional difference in intertidal population when compared with that in subtidal counterparts at 0 h from the two sites) and plastic changes of these genes at each sampling time using a Spearman’s rank correlation. We compared the value with the distribution of values obtained from 1,000 random permutations of the population and treatment group labels in SPSS version 19.0 software (IBM SPSS Statistics). Furthermore, we calculated the evolved change in plasticity values between the intertidal and subtidal populations at each site with respect to the three types of genes, and counted the genes showing increased plasticity during heat stress.

## Results

### Phenotypic Variations

#### Growth

**FIGURE 1:**
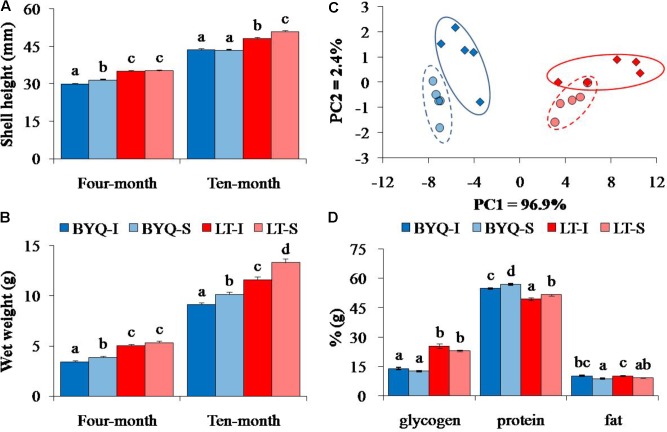
Adaptive divergence in the growth and content metabolites among the four oyster populations. The oysters were collected from the intertidal and subtidal zones of Bayuquan (BYQ) and Laoting (LT). The letters indicate significant differences in multiple comparison (*p* < 0.05). **(A)** Shell height and **(B)** wet weight of 4- and 10-month-old progeny (*n* = 123–150). **(C)** The PCA plot of total protein, glycogen, and fat content of 11-month-old oysters (*n* = 50). **(D)** The content of major nutrients of 11-month-old oysters (*n* = 50).

The Shell height and wet weight of LT oysters were significantly higher than those of 4- and 10-month-old BYQ oysters (*p* < 0.05). Ten-month-old subtidal oysters from LT exhibited higher growth rate than the intertidal counterparts. A similar growth pattern was also detected in BYQ oysters, except the shell height between 10-month-old intertidal and subtidal oysters (**Figures 1A,B**).

#### Metabolites

The PCA analysis of major nutrients (glycogen, protein, and fat) revealed clear differentiations at both latitudinal and vertical levels. The oysters from the two sites were separated by PC1, and PC2 explained the divergence between the intertidal and subtidal populations (**Figure 1C**). The LT oysters have significantly evolved with 80.2% higher glycogen content, but with 10.6% lower protein content when compared with those in BYQ oysters (*p* < 0.05). Furthermore, at both the sites, the intertidal oysters exhibited significantly higher fat content with lower protein content when compared with those of subtidal oysters (*p* < 0.05; **Figure 1D**).

#### Survival Rate

**FIGURE 2:**
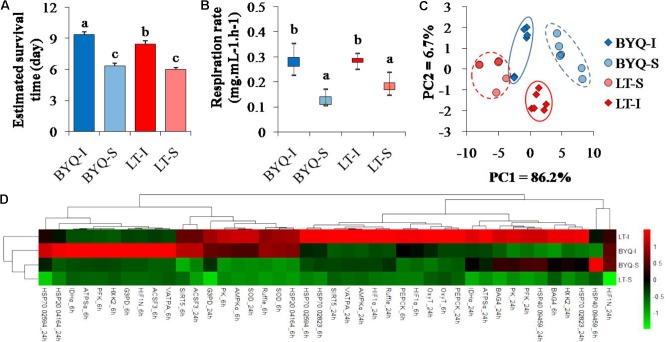
Adaptive divergence in thermal responses at organismal, physiological, and molecular levels among the four oyster populations. The oysters were collected from the intertidal and subtidal zones of Bayuquan (BYQ) and Laoting (LT). The letters indicate significant differences in multiple comparison (*p* < 0.05). **(A)** Survival time of 4-month-old oysters post-acute heat stress of 42°C for 1 h (*n* = 206–326). **(B)** Metabolic rate of 2-year-old oysters at 35°C (*n* = 6). **(C)** The PCA analysis of physiological parameters, including the content of malondialdehyde (MDA), activities of ATPase and superoxide dismutase (SOD) of 10-month-old oysters under heat stress (*n* = 15). **(D)** Hierarchical cluster analysis of relative expression level of the candidate heat-response genes at 35°C (*n* = 15).

The intertidal oysters exhibited significantly higher survival rate post-acute heat stress than the subtidal oysters at both the sites (*p* < 0.05; **Supplementary Figure 3**). Besides, the intertidal oysters in BYQ exhibited higher thermal tolerance than those in LT (**Figure 2A**).

#### Metabolic Rate

The intertidal oysters showed significantly higher oxygen consumption rate than the subtidal counterparts under high temperature at both the sites (*p* < 0.05). However, no difference was observed between the oysters from the two sampling sites at the same vertical level (**Figure 2B**).

#### Physiological Assessment

Two major axes of the PCA analysis explained 92.9% variation in the physiological parameters during heat stress. Furthermore, PC1 clearly separated intertidal oysters from subtidal oysters, and the oysters from each sampling site were identified by PC2 (**Figure 2C**). There were significant differences in the heat responses of physiological parameters between the intertidal and subtidal populations. Further, the dynamic pattern of these indexes between the oysters in BYQ and LT was different (**Supplementary Figure 4**).

#### Gene Expression

The oysters from the same vertical level (intertidal or subtidal) were clustered (**Figure 2D**). Furthermore, most of the heat response genes from the intertidal oysters exhibited higher expressional plasticity than their subtidal counterparts (73.8% in BYQ, 78.6% in LT; **Supplementary Figure 5**).

### Transcriptomic Analysis

#### Summary of RNA Sequencing

The raw data pertaining to the transcriptome of the four collected populations have been submitted to the National Center for Biotechnology Information (NCBI) with the accession code PRJNA407831. We mapped 26,089 unique genes and obtained 566,757,598 reads that passed the machine quality filter with 26,832,278–33,474,390 reads per sample (**Supplementary Table 1**). Furthermore, 17,528 genes passed the statistical filter, and were considered as highly expressed genes for the DAPC analyses (**Supplementary Table 2**).

#### PCA Analysis

**FIGURE 3:**
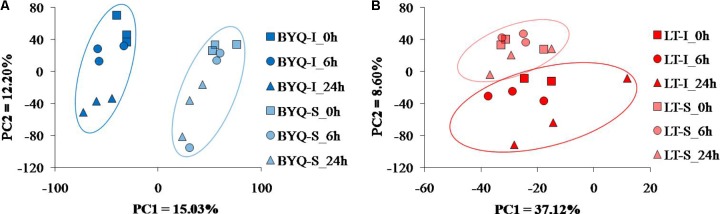
PCA plots of gene expression for the oysters from **(A)** Bayuquan (BYQ) and **(B)** Laoting (LT).

The intertidal and subtidal populations from both the sampling sites were clearly identified by the PCA analysis. For BYQ-I and BYQ-S populations, two major axes of the PCA analysis explained 28.2% of the variation, and clearly separated the intertidal and subtidal oysters from each other by PC1 (**Figure 3A**). For LT-I and LT-S populations, two major axes of the PCA analysis explained 45.7% of the variation, and separated the intertidal and subtidal oysters from each other by PC2 (**Figure 3B**).

#### DAPC for Transcriptional Plasticity

**FIGURE 4:**
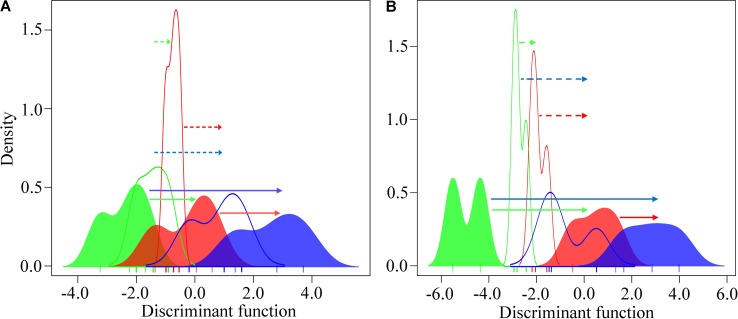
Variation in the genome-wide gene expression plasticity between the intertidal and subtidal oysters from **(A)** Bayuquan (BYQ) and **(B)** Laoting (LT) under heat stress. The arrows indicate the mean transcriptional changes in response to heat stress (dotted line: subtidal, solid line: intertidal). Green, red, and blue indicate sampling times of 0, 6, and 24 h during heat stress.

The whole-genome gene expression profile of the intertidal and subtidal oysters was clearly differentiated in response to heat stress for 6 and 24 h. However, the subtidal oysters presented less pronounced response (**Figure 4**). The intertidal oysters exhibited significantly higher gene expression shifts than the subtidal counterparts at both the sites (*P*_MCMC_ < 0.05; **Supplementary Tables 3A,B**). Furthermore, the divergent expressional plasticity of populations at the same vertical level between the two sites exhibited a similar pattern. The oysters dwelling in BYQ have evolved significantly higher plasticity than the oysters in LT (*p* < 0.05, **Supplementary Figure 6** and **Supplementary Tables 3C,D**).

#### Selection of Three Types of Genes

In total 766 and 785 genes were significantly differentially expressed between the intertidal and subtidal oysters under ambient conditions in BYQ and LT, respectively. Furthermore, 99 genes varied significantly in the oysters from both the sites (**Supplementary Table 4** and **Supplementary Figure 7**). Besides, 952 and 1,717 genes showed significantly plastic changes in response to high temperature among the oysters inhabiting BYQ and LT, respectively; 438 genes concordantly exhibited significantly plastic changes in the oysters from the two sites (**Supplementary Table 5** and **Supplementary Figure 7**). Further, among the 99 evolutionarily divergent genes and 438 concordantly plastic genes, only 37 genes were common to the gene set in which both differentiated significantly (**Supplementary Table 6** and **Supplementary Figure 7**).

#### Direction of Plasticity and Divergence

**FIGURE 5:**
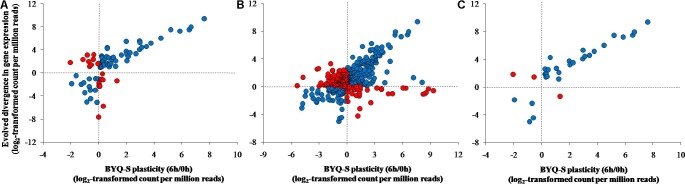
Association between evolved divergence and plastic change in the three types of genes among the subtidal oysters (Bayuquan-subtidal, BYQ-S) exposed to 35°C seawater for 6 h. **(A)** Evolutionarily divergent genes, **(B)** concordantly plastic genes, and **(C)** adaptive plastic genes.

All three types of genes exhibited the same association pattern among the oysters in BYQ, where the direction of evolved divergence was overwhelmingly in the identical direction to that of plastic changes (induced variation in gene abundance of BYQ-S oysters during heat stress for 6 h). Further, 76.5% (65 of 85) of evolutionarily divergent genes showed significantly positive association between evolved divergence and plastic change (**Figure 5A**; ρ = 0.71, *p* < 0.05). Besides, 61.3% (223 of 364) and 90.6% (29 of 32) of all genes exhibited the same positive association with concordantly plastic genes (**Figure 5B**; ρ = 0.45, *p* < 0.05) and adaptive plastic genes (**Figure 5C**; ρ = 0.90, *p* < 0.05), respectively. Furthermore, the plastic change of these three types of genes during heat stress among subtidal population in LT exhibited identical the same direction of evolved divergence between intertidal and subtidal oysters (**Supplementary Figure 8**). In most cases, more than 70% of all genes showed significantly positive association under each condition (**Table 1**).

**Table 1:** Statistics of association between evolved divergence and plastic change in the three types of genes under heat stress.

		*N*p^a^	*N*t^b^	%	ρ	*p*
Evolutionarily	BYQ-S_6h	65	85	76.47%	0.71	3.45E-14
divergent genes	LT-S_6h	61	74	82.43%	0.73	1.64E-13
	BYQ-S_24h	64	86	74.42%	0.66	3.30E-12
	LT-S_24h	53	74	71.62%	0.56	2.03E-07
Concordantly	BYQ-S_6h	221	362	61.05%	0.45	5.94E-20
plastic genes	LT-S_6h	220	315	69.84%	0.52	1.30E-23
	BYQ-S_24h	189	361	52.35%	0.37	5.05E-13
	LT-S_24h	153	296	51.69%	0.12	3.21E-02
Adaptive	BYQ-S_6h	29	32	90.63%	0.90	4.75E-12
plastic genes	LT-S_6h	24	26	92.31%	0.74	1.46E-05
	BYQ-S_24h	26	32	81.25%	0.80	3.33E-08
	LT-S_24h	17	25	68.00%	0.44	2.83E-02

*^a^Genes showed positive correlation between evolved divergence and plastic change. ^b^Total genes in each dataset.*

#### Dynamic Pattern of Increased Plasticity

**FIGURE 6:**
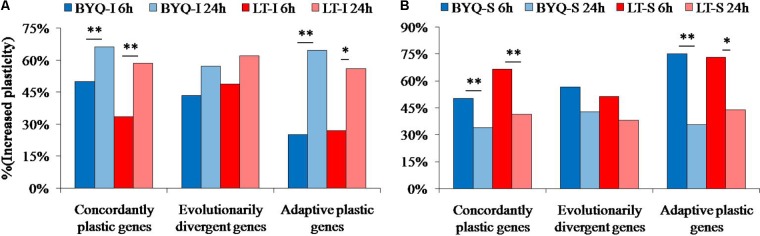
Percent of increased plasticity in the three types of genes among the **(A)** intertidal and **(B)** subtidal oysters under heat stress. Asterisks indicate significant differences (*p* < 0.05).

The oysters from the two sampling sites exhibited a similar dynamic pattern of increased plasticity for all the three types of genes. Furthermore, the occurrence of large-scale increased plasticity in the intertidal oysters (24 h) was significantly delayed when compared with that of the subtidal counterparts (6 h). However, the evolutionarily divergent genes did not exhibit this significantly delayed plastic changes in both intertidal and subtidal oysters (**Figure 6**).

#### Environmental Parameters

The degree of fluctuation in annual temperatures in BYQ is 3.0°C higher than that in LT (**Supplementary Figure 9**). Furthermore, extreme low tides during the warmest month occurring at day time in BYQ was more frequent than those in LT, and the tidal level of BYQ was lower than that of LT (**Supplementary Table 7**).

## Discussion

Adaptive divergence in phenotypic and transcriptomic variations of F_1_ progeny after common garden experiments, among intertidal and subtidal oyster populations from two ecological niches, firmly demonstrated fine-scale genetic differentiation and adaptive evolution in the oysters at both vertical and latitudinal gradients. Even approximately 1-month of planktonic larval stage might incur high gene flow. This vertical adaptive divergence at local scale might be maintained by purifying selection (Sanford and Kelly, 2011). Furthermore, adaptive plasticity will favor high plasticity in rapidly changing environments, such as intertidal zones, by maintaining population size (Fischer et al., 2016; Markov and Ivnitsky, 2016). However, it is at the costs of evolutionary trade-offs between thermal tolerance (enhanced) and growth (constrained).

### Fine-Scale Vertical and Latitudinal Adaptive Divergence

The Pacific oysters from different vertical gradients exhibited distinct phenotypic variations at both the sampling sites. The intertidal oysters showed higher survival rate, metabolic rate, and expressional plasticity under high temperature; however, they exhibited constrained growth. The results elucidate fine-scale adaptive divergence along the sharp vertical gradient at different hierarchical networks from phenotypic (morphological and physiological) to molecular levels, which has been observed in many marine invertebrates (Bozinovic et al., 2011; Somero, 2012; Kenkel and Matz, 2016). Most importantly, it also suggests the recruitment of evolutionarily energetic trade-offs. This adaptive mechanism is prevalent in the marine mollusks, such as eastern oysters, mussels, and snails, inhabiting highly heterogeneous thermal environment (intertidal zone); they are more thermal tolerant, but exhibit slow growth (Bayne, 2004; Petes et al., 2008). Furthermore, it supports the energy balance theory, that is, organisms should maintain homeostasis between the energy for fitness-related functions and the basal maintenance to environmental stressors (Sanford and Kelly, 2011; Sokolova et al., 2012). The theory could well explain the slow growth of intertidal oysters, where most energy was used to adapt to rapidly changing intertidal environments. In addition, the transcriptomic data showed clear vertical differentiation at different tidal heights in both the sampling sites. The whole-genome gene expression profile also revealed clear adaptive divergence between the mussels inhabiting different vertical levels (Place et al., 2012). The results indicate that the environmental selection imposed by rhythmic air exposure and high temperature variation encountered in the intertidal zones potentiates this fine-scale vertical adaptive differentiation.

Further, the oysters also showed a similar pattern of phenotypic variations along the latitudinal gradient. The oysters inhabiting relatively benign environments (LT) exhibited high growth at both the developmental stages and glycogen content in comparison with those of more stressed counterparts (BYQ) at the same vertical level. However, the oysters from BYQ had high protein content and survival rate when exposed to acute heat stress. Our findings further demonstrate latitudinal adaptive divergence and support the evolutionary trade-offs between development and thermal tolerance in the oyster. This is consistent with our previous findings of adaptive divergence between northern and southern oyster populations, that is, oysters in the northern coasts (high environmental variability) are thermal tolerant, but with constrained growth (Li et al., 2018). High growth rate under moderate environment conditions has been reported in the congener eastern oyster and Atlantic silverside (Hice et al., 2012; Burford et al., 2014). The energetic trade-offs among diverse adaptive traits have been observed in different marine ectotherms in response to various stressors (Pörtner, 2010; Pan et al., 2015; Sussarellu et al., 2016). This has been reported in aquatic fishes, as well (Dupont-Prinet et al., 2010; Killen, 2014). The physiological evolutionary trade-offs between oysters at both vertical and latitudinal gradients indicate the phenotypic adaptive mechanisms of this marine invertebrate.

Latitudinal differences were detected in the growth traits and content of metabolites under normal condition, whereas vertical differences were detected in the survival traits, metabolic rate, and expressional plasticity under heat stress. We propose that the thermal stress might play a more important role in shaping vertical adaptive divergence between intertidal and subtidal oysters, while in addition to temperature, other environmental factors might contribute to the latitudinal adaptive differentiation. Furthermore, the opposite direction of marine currents does not strongly facilitate gene flow between these two sites.

### Evolution Toward Adaptive Plasticity

With the establishment of fine-scale vertical adaptive divergence, we further determined the role of plasticity in this evolutionary process. A strong pattern of positive association was observed between evolved divergence and plastic changes for all three types of genes in the two subtidal oyster populations under heat stress. This indicates that the direction of change in gene expression in the subtidal oysters under thermal stress is overwhelmingly in the same direction to that of evolved changes, indicating evolution toward adaptive plasticity (Ghalambor et al., 2015; Fischer et al., 2016). Several studies have observed this positive relationship between the direction of plasticity and evolved divergence of mRNA expression in coastal fishes (Fraser et al., 2014; Shaw et al., 2014) and other marine species by proteomic and physiological analyses (Schaum and Collins, 2014; Makinen et al., 2016). We suggest that this adaptive plasticity can contribute to the adaptive evolution of organisms inhabiting highly heterogeneous environments. In particular, the intertidal oysters have optimized phenotypic integration to achieve optimal fitness by constraining growth and by enhancing thermal tolerance and plasticity, and vice versa in subtidal oysters. The adaptive plasticity will buffer the intertidal populations from extinction and provide long time for evolutionary responses to environmental change (Nadeau et al., 2017). The plastic responses will efficiently predict the direction of subsequent evolutionary processes (Fischer et al., 2016). Further, the intertidal populations inhabiting highly variable environments might have high adaptive potential and weak extinction risk considering the current global warming. The future studies on genetic mechanisms of adaptive plasticity are necessary to deeply understand the relationship between genetic composition and plasticity.

We hypothesized that the evolution of plasticity will be high in the intertidal oysters because of the underlying natural selection that acts strongly on transcripts exhibiting adaptive plasticity. The evolution of magnitude of transcriptomic plasticity was examined and found to be distinctly different among sampled oysters along both vertical and latitudinal gradients. The organisms from high fluctuating environments (intertidal vs. subtidal and northern vs. southern populations) have evolved greater transcriptional plastic changes in response to heat stress. A similar transcriptomic divergence at vertical gradient has been demonstrated in the corals, where the inshore population exhibited enhanced plasticity to adapt to variable environments of inshore zones (Kenkel and Matz, 2016). Furthermore, a similar latitudinal divergence pattern of increased expressional plasticity was observed in other oyster populations dwelling in more heterogeneous northern environment in comparison with that of their southern counterparts (Li et al., 2018). In the present study, the time of large-scale increased plastic changes of the three types of genes in the subtidal oysters was earlier than that in the intertidal populations under high temperature. This indicates that the subtidal oysters are thermal sensitive because the high degree of plastic changes in the organisms under challenging conditions always requires more energy and material costs (DeWitt et al., 1998; Siljestam and Ostman, 2017). The results suggest that the populations from high temporospatial variable climate (especially temperature disturbance) will evolve high plasticity and be less vulnerable to increasing climate change (Nadeau et al., 2017). Our findings provide direct evidence for resource conservation, especially for oysters with low plasticity, in the context of exacerbated global warming. Furthermore, understanding the evolutionary responses can help select suitable genetic recourses with high adaptive potential for the reestablishment of oyster reefs.

Alternatively, littoral amphipods have evolved inverse adaptive strategy (Bedulina et al., 2013). In the species inhabiting upper littoral zone (thermal tolerant), the gene hsp70 exhibited higher constitutive expression, but with lower plasticity when compared with those in its lower littoral congener (thermal sensitive). Investigations regarding the underlying mechanisms of selection for alternative adaptive strategy are required in the future.

### Countergradient Variation

More than 91% concordantly plastic genes did not exhibit significantly evolved divergence between the intertidal and subtidal oysters. This indicates that phenotypic plasticity and evolutionary divergence work orthogonally on different sets of genes. Similar findings have been reported in other marine animals, such as coastal teleost fish (Whitehead and Crawford, 2006; Dayan et al., 2015) and the freshwater fish pike cichlid (Ghalambor et al., 2015). Further, the number of evolutionarily divergent genes showing increased plastic changes did not vary significantly during heat stress. However, the dynamic expression pattern of the other two types of plastic genes was detected in response to high temperature. These results indicate that the evolutionarily divergent genes are independent of thermal stress intensity. The results further support that plasticity and evolutionary divergence work on different functional genes. Furthermore, lower amounts of evolutionarily divergent genes when compared with that of concordantly plastic genes implies that near-perfect plasticity dominates adaptive divergence of the oysters along vertical gradients (Price et al., 2003; Dayan et al., 2015). This suggests that the oysters might have high evolutionary potential to interact with increasing global climate change at transcriptome level. The oyster larvae were proved to have evolved high evolutionary potential in response to three major marine environmental stressors (temperature, salinity, and pH) at proteome level (Dineshram et al., 2016).

## Conclusion

The results of the present study elucidate fine-scale adaptive divergence of the oyster at both vertical and latitudinal gradients using specific phenomic and transcriptomic analyses. (1) The phenotypic mechanisms of evolutionary trade-offs between growth and thermal tolerance, (2) high plasticity in rapidly changing environments, and (3) adaptive plasticity showing the same direction as selection provide insights into the adaptive mechanisms of the oyster dwelling in severe coastal zones. Further studies on the genetic mechanisms of adaptive plasticity are necessary to deeply understand the relationship between genetic composition and plasticity. These patterns provide direct evidence for resource conservation, especially for the oysters with low plasticity, in the context of exacerbated global warming. Furthermore, understanding the evolutionary responses can help select suitable genetic recourses of high adaptive potential for the reestablishment of oyster reefs.

## Data Accessibility

The transcriptome data have been deposited in the Sequence Read Archive (SRA) database with the accession number PRJNA407831.

## Author Contributions

LL and GZ conceived the idea and designed the methodology. LL and WW collected the oysters and oversaw spawning. KS uploaded the RNA-Seq data to the NCBI. AL collected and analyzed the data. AL, LL, and GZ drafted the manuscript.

## Conflict of Interest Statement

The authors declare that the research was conducted in the absence of any commercial or financial relationships that could be construed as a potential conflict of interest.

## Footnotes

1 https://oceandata.sci.gsfc.nasa.gov/
