# Supplementary material for: Transcriptomics and Fitness Data Reveal Adaptive Plasticity of Thermal Tolerance in Oysters Inhabiting Different Tidal Zones
Source: Front Physiol. 2018 Aug 20;9:825. doi: 10.3389/fphys.2018.00825 (PMC6120431; doi:10.3389/fphys.2018.00825)
Supplement: Supplementary file 6 [file Data_Sheet_1.doc]

Supplementary Material

# Transcriptomics and Fitness Data Reveal Adaptive Plasticity of Thermal Tolerance in Oysters Inhabiting Different Tidal Zones

**Ao Li,^1,3^ Li Li,^1,2,5*^ Wei Wang,^1,4,5^ Kai Song,^1,4,5^ and Guofan Zhang^1,4,5*^**

*** Correspondence:**

Guofan Zhang

gzhang@qdio.ac.cn

Li Li

[lili@qdio.ac.cn](mailto:lili@qdio.ac.cn).

**Supplementary Figure 1.** Sampling site map of four wild oyster populations. The oysters were collected from the intertidal and subtidal zones of Bayuquan and Laoting, and translocated to Qingdao for one-generation common garden experiments. The purple lines indicate marine currents during summer. ① Yellow Sea warm current, ② Liaodong Bay coastal current, and ③ Bohai Bay coastal current.

**Supplementary Figure 2.** Experimental design of the present study. Wild oysters were collected from the intertidal (I) and subtidal zones (S) of Bayuquan (BYQ) and Laoting (LT). The F_1_ progeny after one-generation common gardening was used to measure growth rate and content of metabolites (normal condition, light blue); survival rate (42°C, red); metabolic rate, physiological indexes, gene expression, and transcriptome level (35°C, pink).


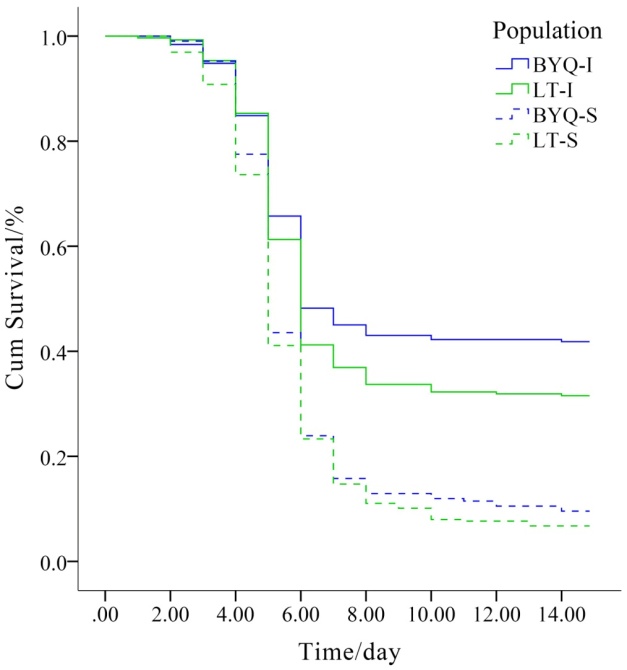


**Supplementary Figure 3.** Cumulative survival rate during recovery after 1 h of acute heat stress. BYQ-I, Bayuquan-intertidal; LT-I, Laoting-intertidal; BYQ-S Bayuquan-subtidal; LT-S, Laoting subtidal.


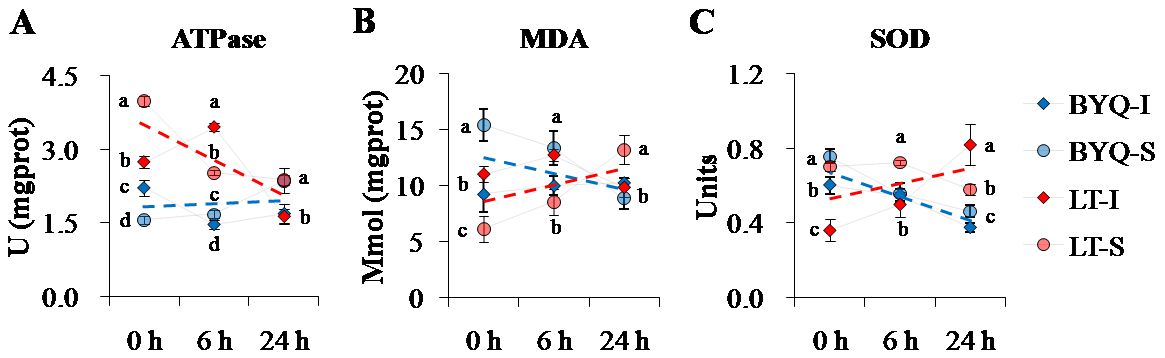


**Supplementary Figure 4.** Thermal responses of (**A**) ATPase activity, (**B**) malondialdehyde (MDA) content, and (**C**) superoxide dismutase (SOD) activity in the four oyster populations under heat stress. Different letters at the same time point indicate significant differences (*p* < 0.05), and error bars represent SDs. BYQ-I, Bayuquan-intertidal; LT-I, Laoting-intertidal; BYQ-S Bayuquan-subtidal; LT-S, Laoting subtidal.


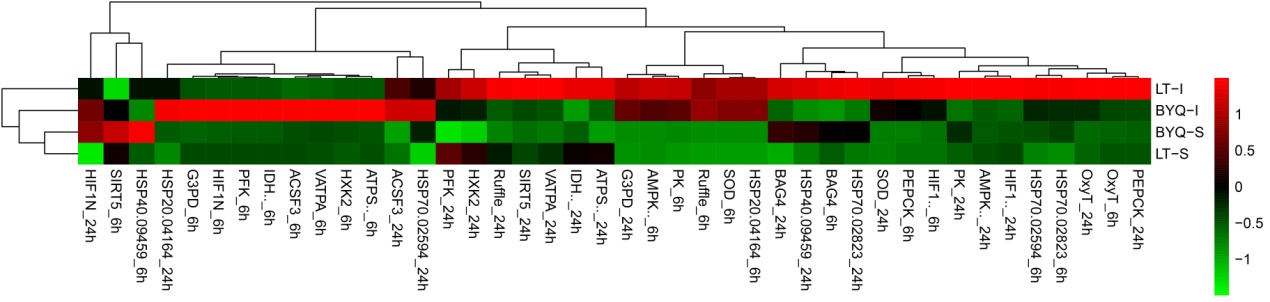


**Supplementary Figure 5.** Absolute value of plastic change of candidate genes during heat stress.


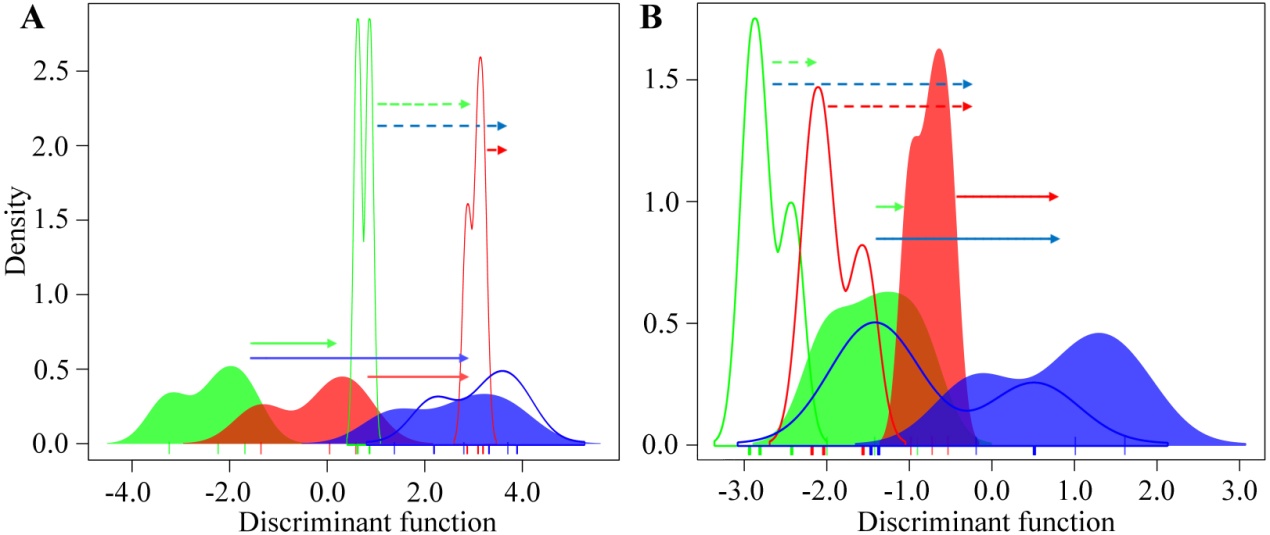


**Supplementary Figure 6.** Variation in the genome-wide gene expression plasticity between Bayuquan (BYQ) and Laoting (LT) oysters at (**a**) intertidal and (**b**) subtidal zones during heat stress. The arrows indicate the mean transcriptional changes in response to heat stress (dotted: LT, solid: BYQ). Green, red, and blue indicate sampling times of 0, 6, and 24 h during heat stress.

**Supplementary Figure 7.** Selection of three type genes. (**a**) Genes differentially expressed between the intertidal and subtidal oysters in Bayuquan (BYQ) (blue) and Laoting (LT) (green), as well as genes that were differentially expressed at both the sites. (**b**) Genes showed significantly plastic changes in BYQ and LT, and concordantly plastic changes at both the sites. (**c**) Genes that were both evolutionarily divergent (yellow) and significantly plastic (pink).

**Supplementary Figure 8.** Association between evolved divergence and plastic change of the three types of genes under heat stress. (**a**, **d**, **g**) Evolutionarily divergent genes, (**b**, **e**, **h**) concordantly plastic genes, and (**c**, **f**, **i**) adaptive plastic genes. BYQ-S, Bayuquan-subtidal; LT-S, Laoting-subtidal.


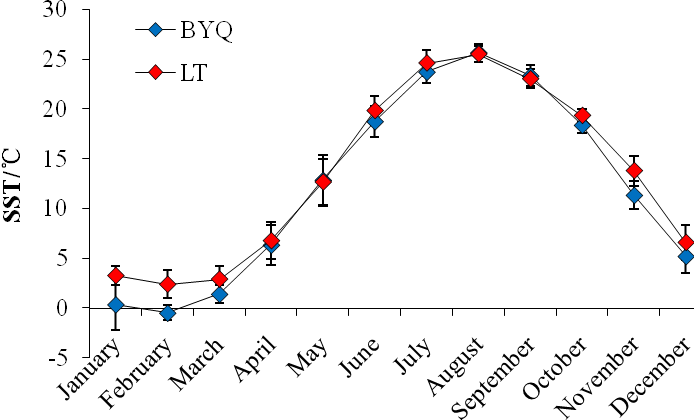


**Supplementary Figure 9.** Satellite remote sensing data of sea surface temperature (SST) within 30 km from the sampling sites (Bayuquan (BYQ) and Laoting (LT)) during the last 18 years (from 2000 to 2017).

**Supplementary Table 3.** *P_MCMC_* values of population-level plasticity divergence between the intertidal and subtidaloysters from Bayuquan (BYQ) and Laoting (LT) during heat stress.

A

| ***P_MCMC_*** | | BYQ-I | | |
| --- | --- | --- | --- | --- |
|  |  | 6 – 0 h | 24 – 0 h | 24 –6 h |
| BYQ-S | 6 – 0 h | 0.48 |  |  |
|  | 24 – 0 h |  | 0.0061 | 0.0011 |
|  | 24 – 6 h |  |  | 0.0018 |

B

| ***P_MCMC_*** | | LT-I | | |
| --- | --- | --- | --- | --- |
|  |  | 6 – 0 h | 24–0 h | 24 – 6 h |
| LT-S | 6 – 0 h | 0.0021 |  |  |
|  | 24 – 0 h |  | 0.0018 |  |
|  | 24 – 6 h |  |  | 0.15 |

C

| ***p_MCMC_*** | | BYQ-I | | |
| --- | --- | --- | --- | --- |
|  |  | 6 – 0 h | 24 – 0 h | 24 – 6 h |
| LT-I | 6 – 0 h | 0.79 |  |  |
|  | 24 – 0 h |  | 0.0064 | 0.00071 |
|  | 24 – 6 h |  |  | 0.0021 |

D

| ***p_MCMC_*** | | BYQ-S | | |
| --- | --- | --- | --- | --- |
|  |  | 6 – 0 h | 24 – 0 h | 24 – 6 h |
| LT-S | 6 – 0 h | 0.05 |  |  |
|  | 24 – 0 h |  | 0.0039 |  |
|  | 24 – 6 h |  |  | 0.1 |

**Supplementary Table 7.** Time of extreme tides and the corresponding tidal level in Bayuquan (BYQ) and Laoting (LT) during the warmest month (August).

| Date | BYQ | | LT | |
| --- | --- | --- | --- | --- |
|  | Extreme tide (Hrs) | Tidal level (cm) | Extreme tide (Hrs) | Tidal level (cm) |
| 1 | 4:52 | 94 | 1:36 | 130 |
| 2 | 5:45 | 106 | 2:42 | 121 |
| 3 | 6:48 | 113 | 3:51 | 111 |
| 4 | 7:50 | 110 | 4:54 | 101 |
| 5 | 8:45 | 101 | 5:45 | 91 |
| 6 | 9:32 | 90 | 6:29 | 85 |
| 7 | 10:14 | 81 | 7:08 | 82 |
| 8 | 10:53 | 75 | 7:44 | 83 |
| 9 | 11:29 | 72 | 8:18 | 85 |
| 10 | 12:06 | 72 | 8:50 | 90 |
| 11 | 12:43 | 75 | 9:23 | 96 |
| 12 | 1:32 | 77 | 9:56 | 103 |
| 13 | 2:06 | 69 | 10:32 | 112 |
| 14 | 2:44 | 64 | 11:10 | 124 |
| 15 | 3:27 | 64 | 0:00 | 124 |
| 16 | 4:17 | 70 | 0:52 | 114 |
| 17 | 5:16 | 78 | 2:04 | 104 |
| 18 | 6:27 | 83 | 3:21 | 93 |
| 19 | 7:41 | 78 | 4:36 | 82 |
| 20 | 8:48 | 66 | 5:40 | 74 |
| 21 | 9:47 | 53 | 6:32 | 70 |
| 22 | 10:39 | 45 | 7:18 | 72 |
| 23 | 11:25 | 44 | 8:01 | 78 |
| 24 | 12:09 | 50 | 8:41 | 88 |
| 25 | 12:51 | 60 | 9:19 | 99 |
| 26 | 1:32 | 62 | 9:55 | 111 |
| 27 | 2:04 | 63 | 10:31 | 125 |
| 28 | 2:37 | 68 | 23:44 | 120 |
| 29 | 3:12 | 78 | 0:00 | 121 |
| 30 | 3:53 | 92 | 0:39 | 116 |
| 31 | 4:43 | 108 | 1:43 | 113 |

**Supplementary Table 1.** Summary statistics of transcriptome mapping database.

**Supplementary Table 2.** Standard transformed expression level of highly expressed genes.

**Supplementary Table 4.** Genes exhibited significant divergence between intertidal and subtidal oysters from BYQ, LT and both sites under normal condition.

**Supplementary Table 5.** Genes exhibited significantly plastic change of oysters from BYQ, LT and both sites during heat stress.

**Supplementary Table 6.** Genes exhibited both evolutionary divergence and concordantly plastic change
